# The COVID-19 Pandemic and Family Violence: Reflecting on Two Years’ Research

**DOI:** 10.1007/s10896-022-00410-9

**Published:** 2022-05-10

**Authors:** Rebecca J. Macy

**Affiliations:** School of Social Work, University of North Carolina at Chapel Hill, 325 Pittsboro Street, CB #3550, Chapel Hill, NC 27599 USA

## A March 2020 Call for Manuscripts

In December 2019, an unknown pneumonia virus emerged in Wuhan, Hubei Providence, China (Centers for Disease Control and Prevention [CDC], 2022). By early 2020, the World Health Organization (WHO), which named the virus “COVID-19,” began to act to address this novel public health threat. On March 11, 2020, the WHO pronounced COVID-19 to be a pandemic, and around the globe countries declared national states of emergencies that included policies to enact masking, social distancing, and “lockdowns” (e.g., quarantines, stay-at-home orders). In addition, to mitigate the spread of the virus and protect healthcare systems from being overwhelmed, many businesses and workplaces, government offices, schools and universities were closed (CDC, 2022).

Closures, social distancing policies, and stay-at-home orders were enacted foremost due to the considerable concerns for health, safety, and lives in the face of a global pandemic driven by a novel virus. Quickly however alarms about the unintended consequences of pandemic mitigation strategies began to emerge. A particularly worrisome set of unintended consequences was increases in and exacerbations of family violence generally, as well as intimate partner violence (IPV), also known as domestic abuse or domestic violence, specifically (Boserup et al., 2020; Bradbury-Jones & Isham, 2020).

Accordingly, on March 27, 2020, the *Journal of Family Violence* (JOFV) issued a call for manuscripts concerning the relationship of COVID-19 to all forms of family violence– including child maltreatment, dating violence, elder mistreatment, and IPV– to generate evidence about the relationships among family violence, the pandemic, and pandemic mitigation strategies. This call was critically important because, on the one hand, mitigation policies were clearly necessary to moderate and manage a worldwide public health crisis. On the other hand, with all forms of family violence potentially worsening because of the pandemic and mitigation strategies, there was also a pressing need to platform the research of scholars who were working to prevent and address family violence in the context of COVID-19.

## Disseminating Family Violence Research Concerning the COVID-19 Pandemic

In the past two years, the March 2020 call led to the online publication of 36 articles concerned with the COVID-19 pandemic and family violence in JOFV. Now, with a considerable body of research developed concerning the pandemic and family violence, the July and the August 2022 journal issues will be dedicated to disseminating much of this work. Overall, these articles span various form of family violence, including child maltreatment (Sinko et al., 2021), domestic violence and IPV (Leigh et al., 2022), elder mistreatment (Liu et al., 2021), and sibling violence (Perkins et al., 2021). Likewise, these articles use a variety of scientific methods, including analytic commentaries (Sharma & Borah, 2020), literature reviews (McNeil et al., 2022), qualitative approaches (Voth Schrag et al., 2022), and quantitative approaches (Spencer et al., 2021). In addition to attending to the connections between family violence and the pandemic in both rural (Moffitt et al., 2020) and urban communities (McLay, 2021; Shariati & Guerette, 2022), these articles were generated from research conducted around the globe, including China (Zhang, 2020), England (Gregory & Williamson, 2021), Norway (Bergman et al., 2021), Portugal (Capinha et al., 2021), and Singapore (Chung et al., 2020). Taken together, this developing body of global evidence provides an opportunity to reflect on and take stock of the connections between family violence and the pandemic, particularly as the worldwide impacts of COVID-19 continue to evolve just as the virus itself does.

## Family Violence in the Context of the Pandemic

### Family Violence, COVID-19, Pandemic Mitigation, and Inequities

Broadly, emerging evidence shows connections between increases in IPV incidence and pandemic mitigation strategies implemented in response to COVID-19 (Piquero et al., 2021). Research also shows the confluence of economic and social disadvantages (e.g., financial and resource insecurity, mental illnesses, overcrowding, unemployment, social isolation), along with other stressors caused by the pandemic, exacerbating or leading to family violence and IPV (Drotning et al., 2022; McNeil et al., 2022). Such findings echo and underscore evidence concerning how the direct impacts of the pandemic were worsened by discrimination, racism, as well as social and structural inequities in peoples’ living and working conditions, as well as in their access to health care (Tai et al., 2021). Likewise, JOFV research suggests that for people without safe homes and for those experiencing disadvantage and discrimination, due to racism and/or as sexual minorities as examples, pandemic mitigation strategies exacerbated family violence and IPV (Drotning et al., 2022; Shariati & Guerette, 2022).

In other words, strategies intended to mitigate the COVID-19 public health emergency did not necessarily work in similar ways across people, communities, and contexts. For people who could retreat to a home free of violence and/or who could continue their employment virtually, the pandemic was a markedly different experience, despite the nonetheless serious challenges, relative to people who did not have safe housing, economic resources, and/or secure employment that allowed them to work in socially distant ways. It is also worth noting here the results from JOFV-published research showing that the pandemic mitigation measures were especially impactful for parents. Particularly concerning were findings of how pandemic-related burnout and stress may have related to harsh parenting and child maltreatment (Chung et al., 2020; Freisthler et al., 2021; Griffith, 2020; Lee et al., 2021).

Similar to how access to and quality of health care varies across people, communities, and context, services for family violence and IPV were not historically constructed to serve all people and communities equitably (Bent-Goodley, 2007; Burman et al., 2004). Accordingly, when people experienced family violence during the pandemic, services and supports to ensure safety and well-being may not have been readily available, particularly for those from marginalized communities and minoritized groups (Drotning et al., 2022; Viveiros & Bonomi, 2020).

#### Summary

The research available thus far highlights the complex and dynamic interrelationships among family violence, discrimination, racism, social conditions, structural inequities, and pandemic stressors, including how inequalities are exacerbated during public health crises, as well as how services and supports to prevent and address family violence are not readily available and welcoming for all.

### Services for Family Violence in the Context of the Pandemic

The emerging evidence concerning responses for family violence highlights how service providers experienced serious challenges in their efforts to provide help to victims of violence and their families during the pandemic (Garcia et al., 2021; Nnawulezi & Hacskaylo, 2021; Wright et al., 2021; Voth Schrag et al., 2022). Research showed that providers experienced increased stress and vicarious trauma while nevertheless endeavoring to deliver services to victims and their families in the context of the pandemic. In addition, providers experienced grave concerns for their own health as they labored to help victims while simultaneously trying to prevent the spread of COVID-19 by enacting mitigation strategies in their own communities and organizations (Nnawulezi & Hacskaylo, 2021; Voth Schrag et al., 2022).

Notably, research showed that victim service organizations dedicated to aiding culturally specific communities, as well as victims from marginalized and minoritized groups, were especially tested by concerns of safety, stress, as well as direct and vicarious trauma (Garcia et al., 2021; Nnawulezi & Hacskaylo, 2021). The significant nature of such challenges was deemed due to many of the concerns noted earlier, including discrimination, racism, and inequities in social and structural conditions. In other words, while helping a victim of family violence may typically be demanding, the nature of the work is further complicated when a victim is also struggling with discrimination, racism, and inequality, particularly in the context of a public health crisis.

Despite challenges and hardships, research also showed that service providers reported developing creative and novel service strategies in the context of the pandemic (Garcia et al., 2021; Nnawulezi & Hacskaylo, 2021; Self-Brown et al., 2020; Wright et al., 2021; Voth Schrag et al., 2022). One key innovation that was hailed to be largely successful was the move to virtual service delivery via technology. In essence, service providers learned that victim services, as well as services to prevent family violence, can be successfully delivered virtually via technology.

At the same time, because not all individuals have access to the necessary technologies to receive services and because technology disparities are unequal across families, communities, and contexts, study findings pointed to the importance of governments, foundations, private donors, and other funders purposively directing resources to ensure that virtual victim services and violence prevention programs are accessible and available to all (Liu et al., 2021; Self-Brown et al., 2020; Wood et al., 2021; Wright et al., 2021). Likewise, research pointed to the importance of active outreach to families and victims who are under-served by health and human service systems generally, as well as victim services and violence prevention programs specifically, for many reasons including though not limited to ablism, discrimination and racism, gender and gender identity, immigration status, justice-system involvement, rurality, as well as a lack of housing, transportation, and/or resources.

In addition, research underscored the need for services, especially safety planning, to emphasize helping families and victims with their fundamental needs for finances, food, housing, and transportation in the context of the COVID-19 public health crisis (Liu et al., 2021; Nnawulezi & Hacskaylo, 2021; Wood et al., 2021). Study findings likewise pointed to the importance of coordinated community responses to family violence. Although family violence prevention and victim service programs may be a key entry point for help seeking, they are often not resourced and structured to offer all necessary remedies and services to prevent and ameliorate violence. Accordingly, programs embedded in a strong network of community connections, including healthcare, housing services, and law enforcement, are best positioned to respond to families’ needs in times of public health crises.

#### Summary

The research available thus far showed that, despite the considerable challenges of the pandemic, providers of victim services and violence prevention programs were able to adapt to find innovative ways, particularly using technology, to deliver services virtually. Overall, the research showing the feasibility and success of virtual services suggests important avenues to aid families and victims with violence even in typical times, outside of public health crises. The research also showed that in future public health crises, violence prevention and victim services will require dedicated resources and supports, as well as strong community connections to ensure that all families can access services, as well as to ensure support for the service providers themselves. Programs dedicated to aiding culturally specific communities, as well as victims from marginalized and minoritized groups, will especially benefit from additional resources and targeted supports.

### Research on Family Violence in the Context of the Pandemic

In addition to addressing the connections between family violence and the pandemic in a variety of ways, the JOFV articles concerning COVID-19 reflect the scientific development of family violence research during the pandemic. For example, in the first several months of the pandemic, the articles published by the journal presented the work of scholars undertaking analytic commentaries and rapid reviews of the literature (e.g., Bracewell et al., 2020; Goodman & Epstein, 2020; Griffith, 2020). These scholars should be commended for their swift, thoughtful efforts to organize extant evidence to help guide family violence practice and policy during a time of global crisis.

As the pandemic continued, researchers found creative ways to collect data as well as conduct studies to inform family violence-related practices and policies efficiently and promptly, including by using data from social media (Freisthler et al., 2021; Lyons & Brewer, 2021; McLay, 2021; Nnawulezi & Hacskaylo, 2021). These studies show that many scholars were able to adapt and innovate their science to generate evidence and research, despite the pandemic.

In addition, researchers have been thoughtfully using administrative data sources to investigate family violence, both over the course of the pandemic and across dynamic changes in pandemic mitigation policies (e.g., Liu et al., 2021; Rebbe et al., 2021). Such investigations provide evidence concerning how and to what extent alterations in the pandemic, as well as mitigation responses to the pandemic related to dynamic changes in and exacerbation of family violence. In turn, evidence from administrative studies can help inform policy and practice both as the pandemic continues in the near-term and in the event of public health crises in the long-term.

Th findings from administrative data sources also underscore the need for greater surveillance data for all forms of family violence. Alarms about the unintended consequences of family violence in the context of the pandemic mitigation strategies emerged early. Nonetheless, with limited information concerning how incidents of family violence may have begun and increased in families and individuals’ lives, communities and governments had little guidance for how best to direct resources to support families and victims. While both global and local surveillance data concerning family violence, including child maltreatment, IPV, and elder mistreatment, would be valuable in the best of times, the pandemic showed how such information is essential in the worst of times. Moreover, surveillance of family violence could valuably include methods beyond the reporting data available from formal authorities (e.g., child protection services, law enforcement), which may not fully reflect the extent and severity of family violence (Capinha et al., 2021; McLay, 2021). While underreporting of family violence is always a concern, pandemic mitigation strategies, like the stay-at-home orders, may have particularly discouraged help-seeking for family violence, especially among children and youth (Capinha et al., 2021; McLay, 2021; Sinko et al., 2021). Ongoing surveys and qualitative research may valuably offer strategies to address the underreporting of family violence to authorities and formal sources of help.

While lauding researchers who were able to begin and/or continue studies during the pandemic for their creativity and resourcefulness, it is also worth considering what research could not or did not happen because of COVID-19. Certainly, because of mitigation strategies and pandemic stressors, the global public health crisis halted or slowed research for many scholars. Accordingly, the violence research community overall, including funders, journal editors, policymakers, as well as researchers, should consider what opportunities might have been foreclosed or missed during the pandemic, as well as what supports are now needed to make up for lost opportunities. Discussions about shoring up family and interpersonal violence research despite COVID-19 could valuably be held at conferences and other scientific meetings, which in turn, could lead to recommendations for future research, as well as the development of direct supports for scholars in the form of dedicated resources, as well as targeted calls for proposals and journal articles. Plans to enact supports to restart research might beneficially focus on bolstering the studies of early-career scholars whose work was likely especially harmed due to the pandemic. Such scientific discussions might also enable opportunities to affirm, build on, and extend the research innovations that emerged during the pandemic. While research opportunities may have been lost in the past two years, creative solutions to keep studies moving forward were also found and are worth retaining, including the increased use of technology to collect data virtually.

#### Conclusion

Encouragingly and despite the challenges of conducting research in the context of a public health crisis, studies concerning family violence continued during the pandemic. In addition, researchers determined novel ways to carry out their research, which will hopefully inform future efforts, both in the context of future public health crises as well as for studies conducted under more typical conditions. Nevertheless, as other scholars have noted (McNeil et al., 2022; Piquero et al., 2021), an empirical understanding of family violence in the context of pandemic is far from complete. Accordingly, by disseminating the research developed in response to the JOFV March 2022 call concerning COVID-19, it is my hope that these articles will help elucidate how and why public health crises can initiate, change, or exacerbate family violence so that we will be better prepared for the future. It is also my hope that research generated during the pandemic will underscore the importance of investigating how family violence relates to inequalities that are with us all the time, including discrimination, racism, as well as in inequitable social and structural conditions. Moreover, as the virus continues to evolve, family violence practices, policies, and research will also need to evolve. For all these reasons, JOFV continues to encourage and welcome research concerned with the pandemic and its relationship to all forms of family violence.

–Rebecca J. Macy, Editor-in-Chief, May 4, 2022
